# Linker-Mediated Inactivation of the SAM-II Domain in the Tandem SAM-II/SAM-V Riboswitch

**DOI:** 10.3390/ijms252011288

**Published:** 2024-10-20

**Authors:** Shanshan Feng, Wenwen Xiao, Yingying Yu, Guangfeng Liu, Yunlong Zhang, Ting Chen, Changrui Lu

**Affiliations:** 1 College of Biological Science and Medical Engineering, Donghua University, Shanghai 201620, China; sandyfss@163.com (S.F.); wenwen_xiao@yeah.net (W.X.); yingyingyu0312@163.com (Y.Y.); zhyl@dhu.edu.cn (Y.Z.); chenting@dhu.edu.cn (T.C.); 2 National Center for Protein Science Shanghai, Shanghai Advanced Research Institute, Chinese Academy of Sciences, Shanghai 201204, China; liuguangfeng@sari.ac.cn

**Keywords:** riboswitch, SAM-II, SAM-V, tandem riboswitch, SHAPE, SAXS

## Abstract

Tandem SAM-II/SAM-V riboswitch belongs to a class of riboswitches found in the marine bacterium ‘*Candidatus* Pelagibacter ubique’. Previous studies have demonstrated that these riboswitches have the potential for digital modulation of gene expression at both the transcriptional and translational levels. In this study, we investigate the conformational changes in the tandem SAM-II/SAM-V riboswitch binding to S-adenosylmethionine (SAM) using selective 2′-hydroxyl acylation analyzed by the primer extension (SHAPE) assay, small-angle X-ray scattering (SAXS), and oligos depressing probing. Our findings reveal that the linker between SAM-II/SAM-V aptamers blocks the SAM response of the SAM-II domain. This result proposes a new mechanism for gene expression regulation, where the ligand-binding functions of tandem riboswitches can be selectively masked or released through a linker.

## 1. Introduction

Riboswitches control gene expression using 5′ untranslated region (UTR) mRNA elements in bacteria, archaea, plants, and fungi [1,2,3,4,5,6,7,8,9]. Specific metabolite binding triggers a series of conformational structure rearranges, modulating the expression of the downstream gene at the transcription or translation level [2,3,10,11,12]. Riboswitches can selectively respond to over 30 different small molecules involved in metabolism or biosynthesis, including ions, vitamins, amino acids, sulfur, and nucleotides [13,14,15]. Over the past 20 years, more than 50 riboswitch classes have been discovered [16,17,18], with S-adenosylmethionine (SAM) riboswitches being the most prevalent [19,20,21]. To date, a total of seven classes of SAM-binding riboswitches have been sorted into the following four major families: SAM-I (including SAM-I, SAM-IV, and SAM-I/IV riboswitches), SAM-II (SAM-II and SAM-V riboswitches), SAM-III, and SAM-VI [22]. Each family of riboswitches senses SAM with distinct conformations to control the expression of downstream genes which involved in methionine or SAM metabolism [23,24,25,26,27,28,29,30].

Canonical riboswitches consist of an aptamer sensor domain followed by an expression platform. Meanwhile, specific riboswitches have been identified that use tandem sensor domains to sense ligands independently, such as glycine riboswitches [31,32,33,34], *glnA* glutamine riboswitches [35,36], and guanidine-II riboswitch [37,38,39,40]. Multiple aptamer riboswitches exhibit a more complicated and precise response to the environmental metabolite concentration [41,42,43,44,45,46,47,48]. Previous studies have shown that tandem SAM-II/SAM-V riboswitches prevail in ‘*Candidatus* Pelagibacter ubique’. It is proposed that the tandem SAM-II/SAM-V riboswitch governs gene expression with different SAM concentrations at both the transcription and translation levels [49]. However, the coordination mechanism of the tandem riboswitches remains unclear.

In this study, we explore the SAM binding mechanism in the tandem SAM-II/SAM-V riboswitch preceding the gene *bhmT* in the marine ‘*Candidatus* Pelagibacter ubique’. Using chemical probing and small-angle X-ray scattering (SAXS), we reconstruct the 3D bead model of the full-length tandem SAM-II/SAM-V riboswitch. We also investigate the SAM binding relationship in the SAM-II domain, linker, and SAM-V domain by a series of antisense DNA oligos depressing probing. Our data suggest that the linker in the full-length SAM-II/SAM-V riboswitch blocks the SAM interaction with the SAM-II domain. This study will benefit the understanding of the subtle mechanisms, design, and application of tandem riboswitches.

## 2. Results

### 2.1. The SAM-II Domain from SAM-II/SAM-V Remains Inactive While the SAM-V Domain Responds to SAM Binding

**Figure 1:**
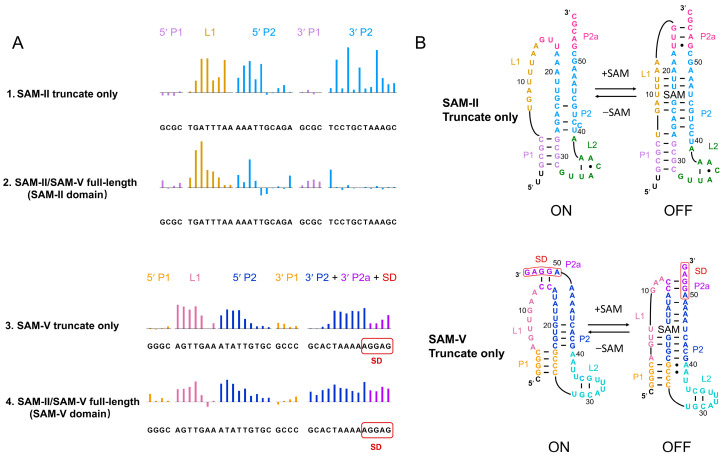
Conformational changes in SAM-II/SAM-V in the truncate only and full-length domains induced by ligand. (**A**) Selective 2′-hydroxyl acylation analyzed by primer extension (SHAPE) reactivity changes in SAM-II/SAM-V in the truncate only and full-length domain under S-adenosylmethionine (SAM) interaction. Colored upward bars represent reduced SHAPE activity upon SAM interaction. The upper two lanes are SHAPE profiles of the SAM-II truncate only (row 1) and the SAM-II domain in full-length SAM-II/SAM-V (row 2). The lower two lanes are profiles of the SAM-V truncate only (row 3) and the SAM-V domain in full-length SAM-II/SAM-V (row 4). Residues are plotted on the *X*-axis. The bar coloring is consistent with the secondary structure model. (**B**) Secondary structure models for the SAM-II and SAM-V truncate only based on the SHAPE analysis. The upper panel indicates the SAM-II truncate-only model; the lower panel indicates the SAM-V truncate-only model. For the SAM-II truncate-only model, P1, L1, P2, P2a, and L2 are plotted in light purple, ginger yellow, sky blue, magenta, and green, respectively. For the SAM-V truncate-only model, P1, L1, P2, P2a, L2, and the SD sequence are shown in orange, pink, navy blue, purple, cyan, and red, respectively.

To compare the conformation of SAM-II/SAM-V in truncate only and corresponding domains in full-length RNA, we employed selective 2′-hydroxyl acylation analyzed by primer extension (SHAPE) assays. The SHAPE data of SAM-II reveal different patterns, indicating different molecular functions performed by the truncate only and corresponding domains in full-length SAM-II/SAM-V. P1 shows minor changes, and L1 and P2 of SAM-II, which contain the SAM-binding site, display a protection pattern under SAM binding in the truncated construct (Figure 1A row 1; Figure S1 row 1 and row 2). In contrast, the SAM-II domain in wild-type full-length SAM-II/SAM-V, including P2, shows little response under SAM presence (Figure 1A row 2). Based on the SHAPE data and crystal structure model (PDB ID: 2QWY), the predicted secondary structure for the SAM-II truncate shows that L1 and P2 form an H-type pseudoknot, the conserved ligand binding sites U10, U11, A20, U21, U22, U46, A47, and A49 locking down SAM (Figure 1B, upper model). Conversely, the SHAPE profile of SAM-V shows almost identical patterns for both the truncate only and corresponding domain in full-length SAM-II/SAM-V (Figure 1A, row 3 and row 4; Figure S1, row 3 and row 4). P1 also displays little changes, L1 and P2 of SAM-V truncate only, and the domain in full-length SAM-II/SAM-V displays a protection pattern (Figure 1A, row 3 and row 4). The predicted secondary structure of the SAM-V truncate only (Figure 1B, lower model) shows the conserved nucleotides 9U, 20G, 45U, 48A, and 49A form the SAM binding core, which is similar to the SAM-II truncate only.

### 2.2. SAXS Show Conformation Changes in SAM-II/SAM-V Riboswitch upon SAM Binding

As previously described, the SHAPE profiles of SAM-II reveal different patterns for the truncate only and corresponding domain in full-length SAM-II/SAM-V. To further investigate the conformation of full-length SAM-II/SAM-V, we employed small-angle X-ray scattering (SAXS) to study the ligand binding dynamics.

**Figure 2:**
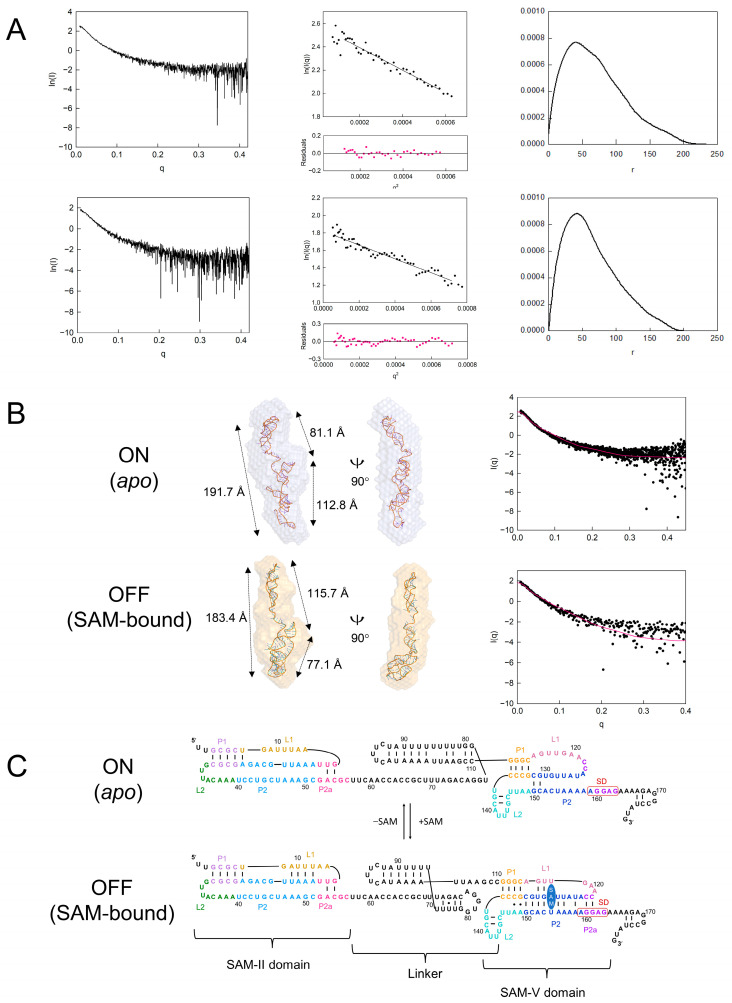
SAM modulates conformational rearrangements of the full-length tandem SAM-II/SAM-V riboswitch. (**A**) Scattering profiles (left panel), Guinier plot (middle panel), and pair distribution functions (*P (r)*) analysis (right panel). The *apo* (upper row series panels) state measure of the radius of gyration (*R*_g_) is ~59.9 Å and the maximum diameter of the particle (*D*_max_) is 233. The SAM bound (lower row series panels) measure *R*_g_ is ~53.1 Å, and *D*_max_ is 200. (**B**) Atomic models of the full-length tandem SAM-II/SAM-V riboswitch superimposed with SAXS-derived bead models. The top model for the *apo* (ON) state is shown in slate; the bottom model for the SAM-bound (OFF) state is shown in bright orange. Atomic model prediction and 3D structure reconstruction were performed using COOT and RNAComposer online servers, which are described in the Material and Methods Section. In the *apo* (ON) state, the dimensions for the top L2 and bottom tail, top L2 and linker loop, and linker loop and bottom tail are 191.7 Å, 81.1 Å, and 112.8 Å, respectively. In the SAM-bound (OFF) state, the dimensions for the top L2 and bottom tail, top L2 and linker loop, and linker loop and bottom tail are 183.4 Å, 115.7 Å, and 77.1 Å, respectively. The theoretical scattering curve (red) derived from the predicted full-length SAM-II/SAM-V atomic model is mapped with experimental SAXS data plotting by CRYSOL, with *apo*: *χ*^2^ = 2.58 and SAM-bound: *χ*^2^ = 2.11. (**C**) Sequence and secondary structure model of the full-length tandem SAM-II/SAM-V riboswitch studied by SHAPE and small-angle X-ray scattering (SAXS) data. The upper model indicates the *apo* (ON) state and the lower model indicates the ligand-bound (OFF) state. Coloring in SAM-II and SAM-V domains is consistent with the legend in Figure 1B. The linker is colored black, and the SAM ligand is oval-shaped in blue.

We determined the 3D model of the full-length SAM-II/SAM-V in both *apo* and SAM-bound states. The monomeric scattering data were confirmed by size-exclusion chromatography. Using 2.5 mM SAM to trigger the folding reaction (consistent with size-exclusion and SHAPE experiments), we obtained a radius of gyration (*R_g_*) of ~59.9 Å from Guinier fit and maximum dimension (*D*_max_) of 233 from paired-distance distributions (*P(r)*) for *apo* full-length SAM-II/SAM-V. For the SAM-bound state, we observed a smaller *R_g_* from Guinier fit of ~53.1 Å and a *D*_max_ of 200 from *P(r)* (Figure 2A).

Based on the SAXS data, we calculated the bead models of the full-length SAM-II/SAM-V. As there is no dissolved full-length SAM-II/SAM-V riboswitch crystal structure to date, we predicted, reconstructed, and fit the calculated envelopes with bead models as previously described. The predicted atomic models and our SAXS data-derived bead models superimpose well in angles and dimensions, and the theoretical scattering curve of our predicted atomic models agrees with the SAXS data (Figure 2B; *apo*: *χ*^2^ = 2.58, SAM-bound: *χ*^2^ = 2.11). The overall architecture of the *apo* full-length SAM-II/SAM-V riboswitch adopts a vertically twisted W-shaped conformation. The dimensions of the L2 and bottom tail, top L2 and linker loop, and linker loop and bottom tail are 191.7 Å, 81.1 Å, and 112.8 Å respectively (Figure 2B upper model). The secondary structure of the *apo* full-length SAM-II/SAM-V riboswitch derived from the atomic model (Figure 2C upper model) shows that the linker forms a stem loop connecting the SAM-II and SAM-V domains.

The SAM-bound state model displays a shorter rod shape. The relative dimensions of the top L2 and bottom tail, top L2 and linker loop, and linker loop and bottom tail are 183.4 Å, 115.7 Å, and 77.1 Å respectively (Figure 2B lower model). The secondary structure of the SAM-bound full-length SAM-II/SAM-V riboswitch derived from the atomic model (Figure 2C, lower model) shows that a new stem loop forms in the linker, and SAM is proposed to be located in the ligand binding core of the SAM-V domain.

### 2.3. The SAM-V Domain and Linker Influence the Conformation of the SAM-II Domain

**Figure 3:**
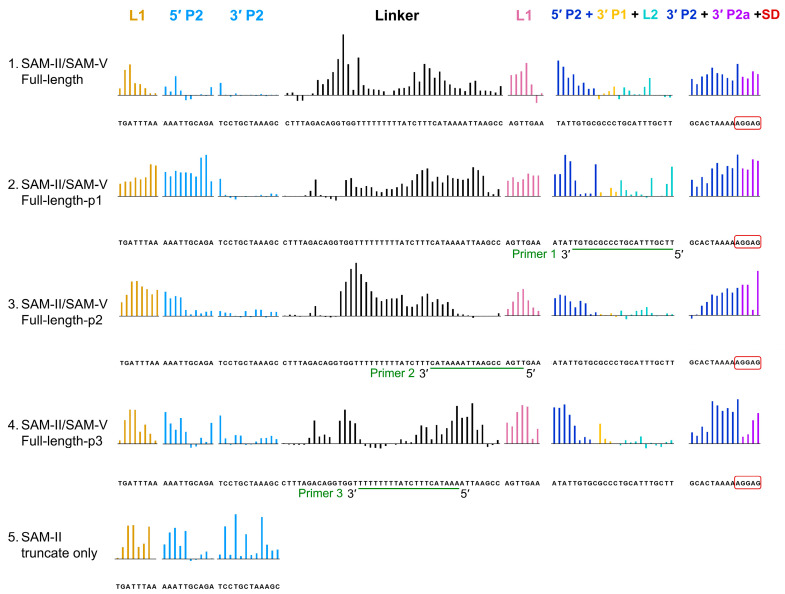
SHAPE analysis of full-length tandem SAM-II/SAM-V riboswitch conformational changes under SAM binding and anti-oligonucleotides interference. Colored upward bars represent reduced SHAPE activity upon SAM binding, with or without primer interaction. Row 1: control reaction without anti-oligonucleotide interference. Row 2: primer 1 pairing with SAM-V 3′-end of 5′ P2, 3′ P1, and L2. Row 3: primer 2 pairing with 3′-end of the linker, 5′ P1, and 5′-end of SAM-V L1. Row 4: primer 3 pairing with U83-U90 poly (U) of the linker. Row 5: SHAPE profiles of SAM-II truncate only. The coloring and motif annotations are consistent with Figure 2C.

To determine the relationship between the conformation of SAM-II and other domains in full-length SAM-II/SAM-V, we employed three antisense DNA oligos distributed in the downstream domain to monitor the potential effects of the linker and SAM-V domain. The primer pairing positions are shown in Figure 3 and Figure S2. Each DNA primer results in different SHAPE reactivity outside the primer complementary region.

The control without primer (Figure 3 row 1) and random primer (Figure S2 row 2) interference resemble the SHAPE profile of full-length SAM-II/SAM-V (Figure 1A and Figure S2), L1 and P2 of the SAM-II domain show little response to the SAM interaction, while ligand binding significantly reduces the flexibility of L1 and P2 in the SAM-V domain, which coincides with the SAM-V truncate-only data (Figure 1A row 2 and 4). The linker of full-length SAM-II/SAM-V responds the most upon SAM binding, especially between C68 and C110. Primer 1 (Figure 3 row 2) pairs with the 5′ P2 and 3′ P1 of the SAM-V domain, outside the SAM binding core. The SAM binding core (L1and P2) and SD of the SAM-V domain, as well as L1 and 5′ P2 of the SAM-II domain, respond to the SAM interaction and display a protected pattern, while 5′ P2 shows little response to SAM. Compared with the control, the sequence upstream of U82-U90 poly (U) in the linker loses the ligand response. Primer 2 pairs with the 3′-end of the linker, 5′ P1, and 5′-end of L1 in the SAM-V domain (Figure 3 row 3). L1, P2, and SD of the SAM-V domain, as well as L1 and P2 of the SAM-II domain, show reduced flexibility. The U82-U90 poly (U) in the linker exhibits a stronger protected pattern compared with the control. Primer 3 (20 nt) (Figure 3 row 4) pairing U83-U90 poly (U) and its downstream sequence in the linker, L1, and P2 of the SAM-V domain display a similar protected SHAPE pattern as the control. L1 and P2 of the SAM-II domain also exhibit a protected pattern, which resembles the SHAPE profiles of SAM-II truncate only (Figure 3 row 5).

In conclusion, our SHAPE analysis shows that the inactivation of the SAM-II domain is caused by its downstream sequences. These sequences disrupt the secondary structures of the linker from C68 to C110, as well as the 5′ P2 and 3′ P1 regions of the SAM-V domain, which are outside the SAM-binding core. Both of these disruptions can restore the SAM interaction of the SAM-II sequence.

## 3. Discussion

Riboswitch crystallization in the ligand-free state remains highly challenging because of its highly flexible conformation. To investigate the overall conformation of the full-length SAM-II/SAM-V riboswitch with or without SAM, we combined powerful small-angle X-ray scattering (SAXS) with selective 2′-hydroxyl acylation analyzed by primer extension (SHAPE), which allowed us to observe local conformational changes in the riboswitch.

Through SHAPE probing analysis, we characterized the conformation of SAM-II and SAM-V truncate only and full-length SAM-II/SAM-V. Our SHAPE data of SAM-II and SAM-V truncate only display a consistent pattern with previous studies [29,49], especially the protection pattern of L1 and P2, which contain the SAM binding site (Figure 1A row 1 and 3), confirming that SAM-II and SAM-V truncate independently interact with SAM. The SHAPE data of the full-length SAM-II/SAM-V riboswitch exhibits a more complex scenario. We observed a similar protection pattern between the SAM-V domain in the full-length and SAM-V truncate only, indicating that the SAM-V domain maintains its independent interaction with SAM (Figure 1A, rows 3 and 4). The linker of full-length SAM-II/SAM-V also shows a wide response to the SAM interaction (Figure 3, row 1). In contrast to SAM-II truncate only, P2 of the SAM-II domain is insensitive to the SAM interaction (Figure 1A, rows 1 and 2). Previous studies [49] observed similar results using in-line probing, coinciding with our results. However, this conclusion remains somewhat puzzling since we did not understand why a well-defined RNA aptamer (SAM-II) can somehow lose its SAM-binding ability. In our study, we employed various annealing methods to ensure homogeneity and improve consistency. Both our SAXS and the SHAPE results indicated that they correspond to a single bond and apo structure. Between the truncated and WT constructs, SAM-V behaves almost the same, while SAM-II behaves vastly differently. Previous studies [49] have also arrived at a similar conclusion. At this point, we do not fully understand the structure or function of the SAM-II domain. We hope to design a study to investigate this domain in the near future.

Tandem riboswitches typically possess multiple aptamers that interact allosterically, forming regulatory logic gates. For instance, the binding of one ligand can promote the binding of another, thereby enhancing regulatory efficiency [34,50,51]. Additionally, tandem riboswitches can bind different ligands to create antagonistic interactions, enabling more precise regulation. Because of the synergistic nature of tandem riboswitches [52,53], we suspect that the downstream sequence of the SAM-II domain in the full-length SAM-II/SAM-V riboswitch may impact P2 of the SAM-II domain.

In our SAXS study, we used 2.5 mM SAM, which is about 20 times the *K*_D_ (~120 uM [49]), to allow the reaction to proceed adequately. Our bead models of the full-length SAM-II/SAM-V riboswitch change from a vertically twisted W-shaped conformation to a more compacted rod-shaped conformation in the presence of SAM. Compared with the *apo* state and SAM-bound state of our full-length SAM-II/SAM-V bead model, the radius of gyration (*R_g_*) decreases from ~59.9 Å to ~53.1 Å. Although no research has reported relative diameters of the *apo* SAM-V riboswitch and the linker at present, *metX* SAM-II riboswitch from the Sargasso Sea metagenome displays a similar change, decreasing from ~21.5 Å to ~19.5 Å under the SAM interaction [54]. The 3D models reveal that the SAM-V domain compresses and adopts a SAM-bound conformation, which is consistent with previous research [26]. The linker and a new hairpin shorten the distance between SAM-II and SAM-V. L2 of the SAM-II domain extends longitudinally and adopts an OFF-like before and after the SAM interaction. These results also suggest that the SAM-II domain is not sensitive to SAM, which is consistent with a previous study [49]. The predicted changes in the linker and SAM-V domain derived from the SHAPE results fit well with the bead models (Figure 2B, CRYSOL *apo*: *χ^2^* = 2.58, SAM-bound: *χ^2^* = 2.11). Because of the limited SAXS resolution, we were unable to observe changes in the SD sequence or AUG within the bead models. The SAXS results serve as an auxiliary to demonstrate that our predicted secondary structure aligns well with the SHAPE results.

For high-resolution studies of conformational dynamics, time-resolved SHAPE, time-resolved hydroxyl radical footprinting, or cryo-electron microscopy (cryo-EM) are ideal choices. Time-resolved SHAPE relies on rapid reagent modification. By initiating RNA folding with the addition of a ligand and the modification, researchers can capture structural snapshots at one-second intervals, observing RNA folding mechanisms at single-nucleotide resolution [55,56]. Time-resolved hydroxyl radical footprinting involves generating hydroxyl radicals, which cleave the RNA backbone at solvent-accessible sites. The cleavage pattern reveals the RNA’s tertiary interactions and RNA–protein contacts. Using synchrotron X-ray beam exposure to generate hydroxyl radicals allows for millisecond-resolution snapshots of RNA folding and interactions [57,58]. Cryo-EM is also used to visualize the structures of biological macromolecules, capturing multiple conformational states in their native environment and studying dynamic processes within cells [25,59].

Riboswitches do not maintain a fixed structure but a dynamic one. All domains and peripheral sequences contribute to the dynamics of the physiological functions of the riboswitch. Previous studies mainly focused on individual domains and their functions. In this study, we used DNA oligos to disrupt non-aptamer regions (linker and SAM-V domain) of the riboswitch systematically to see how they contribute to the dynamics. Our results suggest that sequences outside the SAM binding core of the SAM-V domain can influence the SAM interaction of the SAM-II domain (Figure 3, row 2). Although the SAM-II domain cannot totally restore the SAM interaction pattern of P2, this result suggests SAM-V domain adopts another mechanism to interfere with the SAM-II domain. Our SHAPE data also show that primers 2 and 3, which mainly pair with the linker sequence, enable P2 of the SAM-II domain to significantly restore the interaction with SAM, displaying a pattern similar to SAM-II truncate only. The linker of the full-length SAM-II/SAM-V riboswitch is predicted to form a hairpin before the U82-U90 poly (U) sequence [49], similar to the expression platform of SAM-I [30,60,61]. This suggests that the SAM-II domain and linker may compose a complete aptamer domain and expression platform, affecting the transcription of the downstream SAM-V domain and protein expression. For the linker, our results, including those from previous studies [49], reveal that the presence or absence of the linker does not affect the structure of the downstream SAM-V domain or its ligand binding. However, once the linker is present, it influences the ligand binding of the SAM-II domain. Previous research has shown that the SAM-II domain, when connected to the linker and the SAM-V domain, does not bind SAM [49]. Some regions that DNA anti-oligos paired with still show high reactivity, which may be due to the following reasons: incomplete hybridization with the anti-oligonucleotides; steric effects causing hybridized nucleotides to remain accessible to the SHAPE reagent; or reverse transcriptase pausing induced by intrinsic complex secondary or tertiary structures [62,63,64].

Neither we nor previous studies have observed any synergistic effects in vitro. Perhaps this effect does exist in vivo, with some co-factors or proteins bonding to the riboswitch as a different ligand. We speculate that in vitro studies cannot fully answer this question since allosteric effects, if they exist, require additional molecular ligands to activate the SAM II domain, as we demonstrate with the linker disruption experiments.

Coincidentally, our group is also interested in synthetic biology applications. SAM II/V provides us with a new way to regulate the recombinant expression of target proteins. Since we do not fully understand its physiological function, at this point we cannot use it in its WT form. However, by incorporating a different aptamer, we can create a three-factor regulator that responds to either of the three different ligands (ligands 1 and 2 and a DNA oligo). Additionally, understanding peripheral sequences and their regulatory roles, such as linkers that can influence the functions of these two domains, allows us to leverage this knowledge when designing artificial riboswitches that function in certain specific environments such as high or low temperatures or salt conditions in different chassis, as required by synthetic biology applications. This finding enables us to create a three-factor logical gate for expression regulation.

A previous study shows that nucleotides in the L1 loop form base triplets with the major groove face of the P2b helix, directly participating in SAM binding [29]. However, SAM II behaves vastly differently in SAM II/V, as shown in this study. We have not performed mutations in SAM II/V to induce changes in its conformation. The intracellular variables that contribute to the conformational stability of SAM-II remain unclear. However, we suspect that under physiological conditions, the SAM-II domain, the SAM-V domain, and the linker or peripheral sequences may be influenced by an unknown secondary factor. We would like to investigate this in a separate study.

## 4. Materials and Methods

### 4.1. RNA Synthesis

Three constructs including full-length wild-type tandem SAM-II/SAM-V riboswitch RNA, SAM-II truncate-only RNA, and SAM-V truncate-only RNA from *Candidatus* Pelagibacter ubique were inserted into the pUC57 plasmid under the control of T7 RNAP promoter and amplified by PCR. RNA oligonucleotides used in this study were prepared by in vitro transcription using the appropriate DNA templates and T7 RNA Polymerase. The RNA products were purified by 6% denaturing gel electrophoresis as described [65,66,67]. The target bands were excised and eluted into double-distilled water. Subsequently, the product samples were concentrated and flash-frozen in liquid nitrogen. SAM (RHAWN^®^, Shanghai, China) was prepared as a 100 mM stock in pure water at pH 6.0–6.5. Both product samples and SAM stock were stored at −80 °C before use.

### 4.2. SEC-SAXS Data Collection and Analysis

SEC-SAXS data collection was performed at beamline BL19U2, National Facility for Protein Science Shanghai (NCPSS), and Shanghai Synchrotron Radiation Facility (SSRF). The column used in SEC-SAXS was Superdex 200 Increase 10/300 GL (Cytiva, Marlborough, MA, USA). The column was pre-equilibrated with buffer A containing 25 mM Tris-HCl, pH 7.5, 40 mM NaCl, and 10 mM MgCl_2_, with or without 2.5 mM SAM. Full-length tandem SAM-II/SAM-V riboswitch RNA was dissolved in buffer A at a concentration of 6–8 mg/mL. Subsequently, RNA samples were refolded by heating up to 65 °C for 12 min, followed by the addition of SAM to a final concentration of 2.5 mM. After incubation at 37 °C for 5 min, the samples were cooled on ice. Then, 0.1 mL refolded RNA was automatically injected into the SEC column at room temperature. The measured q-range was ~0.1–4 nm^−1^. The scattering data from the samples were subtracted by the matching buffer background using the software package BioXTAS RAW version 2.1.1 [68]. The data were then input to a set of programs in PRIMUS version 3.2 [69] from ATSAS version 2.8.0 suite [70] to perform the ab initio shape determination. The parameters *R*_g_ and *D_max_* were measured by the program GNOM version 4.6 [71]; DAMMIF Version 1.1.2 [72] was used to generate 20 independent reconstructions, and one averaged low-resolution 3D reconstruction was generated by DAMAVER Version 5.0 [73]. The atomic models of the full-length tandem SAM-II/SAM-V riboswitch were reconstructed by COOT version 0.9.8.92. The 3D predicted structure reconstructions were based on SAXS, SHAPE data, and crystal structure (PDB ID: 2QWY, 6FZ0). The 3D predicted structure reconstructions of the linker were performed using the RNAComposer online server version 1.0 (http://rnacomposer.ibch.poznan.pl/ (accessed on 7 April 2024) [74,75]. The bead models were superimposed onto the predicted structure model using the SUPCOMB version 2.8.0 program [76]. Finally, fitting verification with the theoretical scattering curves was performed using CRYSOL version 2.8.3 [77].

### 4.3. SHAPE Probing Assays

In the SHAPE probing assay, the 177-base full-length tandem SAM-II/SAM-V, SAM-II truncate, and SAM-V truncate RNA sequences were flanked with the SHAPE cassette sequence, and RNA samples were prepared as previously described [78,79,80]. The refolding process was consistent with the SEC-SAXS analysis previously described. First, 1 μL 100 mM 1-Methyl-7-nitroisatoic anhydride (1M7) reagent stock was mixed with 9 μL refolded solution (~10 μM RNA), incubated on ice for 3 min and 30 °C for 10 min. The mixture was then pipetted into the 80% ethanol quench buffer. After 45 min of incubation at −80 °C, the solution was spun at 13,000× *g* and recovered in 10 μL double-distill water. Reverse transcription was initiated by annealing the recovered RNA to the 5′ 6-FAM headed DNA primer. A total 20 μL mixture (1 × HiScript III Buffer and 10 units HiScript III Reverse Transcriptase (Vazyme Biotech Co., Ltd., Nanjing, China), 0.5 mM dNTPs) was incubated at 50 °C for 30 min. The reaction was quenched in 250 mM NaOH at 95 °C for 5 min and neutralized by adding 29 μL acid stop mix. The resulting cDNA products were sequenced by short tandem repeat (STR) capillary electrophoresis (CE) analysis (Sangon Biotech, Shanghai, China). Raw data from the CE analysis were integrated and sequence-fitted using the ShapeFinder version 1.0 program package [81]. Nucleotides (C4-U7, A20, A49 for SAM-II truncate; G3, U33-U34, A43 for SAM-V truncate; C24-G26 for full-length tandem SAM-II/SAM-V) that were similarly modified throughout all experiments were used for normalization and scaling in Excel as previously described [30,67,82]. Extreme signals, possibly induced by pausing, were manually excluded from the data. The secondary structure models of the SAM-II and SAM-V truncates were predicted based on the SHAPE activities and the crystal structure models of homologous sequences (PDB ID: 2QWY, 6FZ0).

### 4.4. Antisense DNA Oligos Annealing Analysis

First, 10 μM of the full-length tandem SAM-II/SAM-V riboswitch RNA was annealed by 3 antisense DNA primers (molar ratio 1: 25~1: 30), the primer being 20 nt in length. Then, the samples were treated with or without 2.5 mM SAM at 37 °C. The following analysis was consistent with the SHAPE analysis previously described.

The antisense DNA oligos sequences are as follows:

Primer 1: 5′-AAGCAAATGCAGGGCGCACA-3′ (pairing position: 128–147).

Primer 2: 5′-ACTGCCCGGCTTAATTTTAT-3′ (pairing position: 98–117).

Primer 3: 5′-TTTATGAAAGATAAAAAAAA-3′ (pairing position: 83–102).

Random primer: 5′-AACCATCTTTCGGCGGACGCG-3′ (no pairing).

## 5. Conclusions

In this study, the tandem riboswitch demonstrates that the linker affects the upstream riboswitch and ensures the functionality of the downstream riboswitch. This result enables flexible regulation of riboswitches and provides new theoretical support for applications in biosensors, biosynthesis, metabolic pathway analysis, and AI design of riboswitches [83,84,85,86].

In conclusion, the SAM-II domain of the full-length SAM-II/SAM-V riboswitch is insensitive to the SAM interaction, and the linker plays the main role in depressing the SAM interaction of the SAM-II domain. This study sheds light on the subtle mechanism, design, and application of tandem riboswitches.

## Data Availability

The original contributions presented in this work are included in this article; further inquiries can be directed to the corresponding author.

## Supplementary Materials

The following supporting information can be downloaded at https://www.mdpi.com/article/10.3390/ijms252011288/s1.
