# Supplementary material for: Linker-Mediated Inactivation of the SAM-II Domain in the Tandem SAM-II/SAM-V Riboswitch
Source: Int J Mol Sci. 2024 Oct 20;25(20):11288. doi: 10.3390/ijms252011288 (PMC11508383; doi:10.3390/ijms252011288)
Supplement: Supplementary file 1 [file ijms-25-11288-s001.zip › ijms-3251903-supplementary.pdf]

Supplementary Materials

Linker-Mediated Inactivation of the SAM-II Domain in the Tandem SAM-II/SAM-V Riboswitch

Shanshan Feng [1], Wenwen Xiao [1], Yingying Yu [1], Guangfeng Liu [2], Yunlong Zhang [1], Ting Chen [1] and Changrui Lu [1,*]

1 College of Biological Science and Medical Engineering, Donghua University, Shanghai 201620, China; sandyfss@163.com (S.F.); wenwen_xiao@yeah.net (W.X.); yingyingyu0312@163.com (Y.Y.); zhyl@dhu.edu.cn (Y.Z.); chenting@dhu.edu.cn (T.C.)
2 National Center for Protein Science Shanghai, Shanghai Advanced Research Institute, Chinese Academy of Sciences, Shanghai 201204, China; liuguangfeng@sari.ac.cn
* Correspondence: crlu@dhu.edu.cn; Tel.: +86-21-67792740

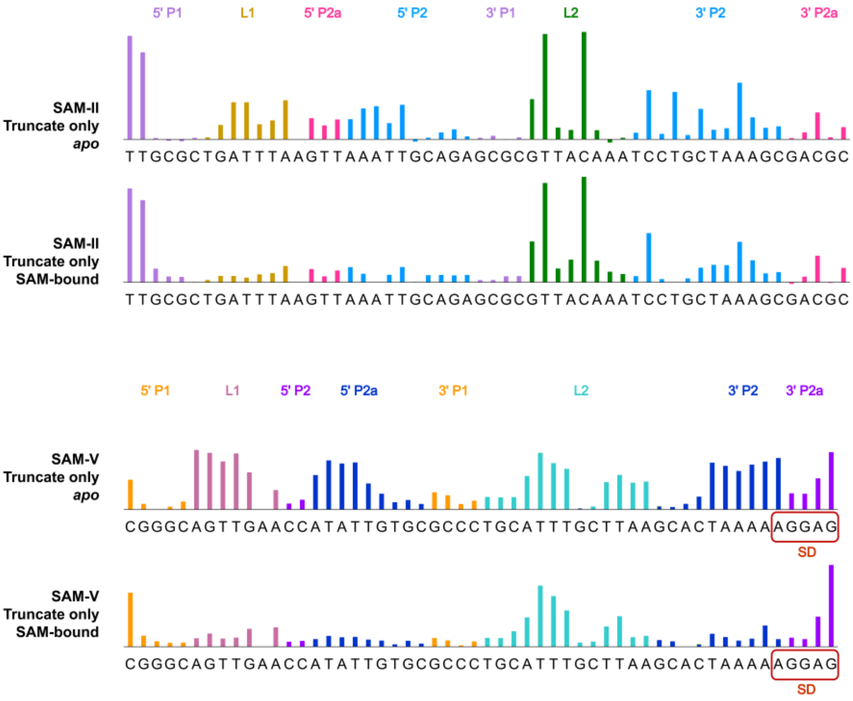

Figure S1. SHAPE analysis of SAM-II/SAM-V in truncate only under the *apo* and SAM bound states. The upper two lanes represent the SHAPE profile of *apo* state (row 1) and ligand-bound state (row 2) for SAM-II truncate only. The lower two lanes represent profile of the *apo* state (row 3) and the ligand bound (row 4) for SAM-V truncate only.

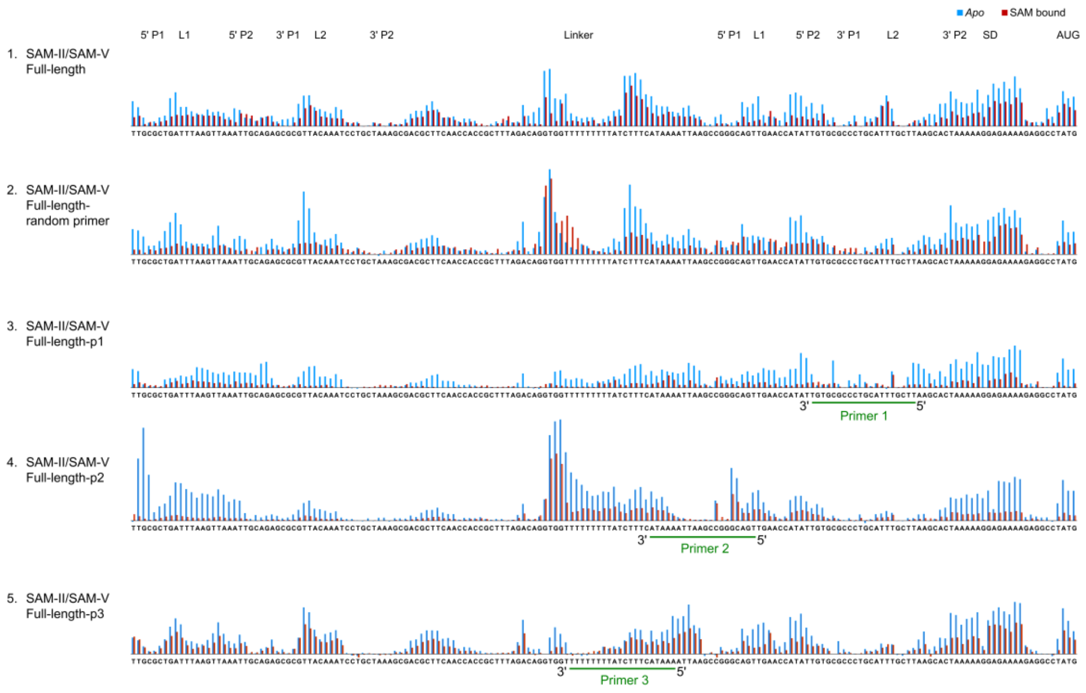

Figure S2. SHAPE analysis of full-length tandem SAM-II/SAM-V riboswitch conformational changes under SAM binding and anti-oligonucleotides interference. Blue bars indicate the *apo* state and red bars indicate SAM bound state. Green horizontal lines indicate the primer positions. Row 1: control reaction without anti-oligonucleotide interference. Row 2: random primer that has no pairing with full-length tandem SAM-II/SAM-V riboswitch. Row 3: primer 1 pairing with the SAM-V 3′-end of 5′ P2, 3′ P1, and L2. Row 4: primer 2 pairing with 3′-end of linker, 5′ P1, and 5′-end of SAM-V L1. Row 5: primer 3 pairing with U83-U90 poly-U of linker. Positive bars indicate reduced SHAPE activity upon SAM interaction and anti-oligonucleotide interference.
